# The Influence of Carbon Sources on the Microstructures of In Situ-Synthesized TiC in Al Melts

**DOI:** 10.3390/ma15134610

**Published:** 2022-06-30

**Authors:** Haimin Ding, Jiangmin Wu, Haoran Jia, Fang Liu, Jinfeng Wang

**Affiliations:** 1 School of Energy, Power and Mechanical Engineering, North China Electric Power University, Baoding 071003, China; dinghaimin@ncepu.edu.cn (H.D.); 220192224004@ncepu.edu.cn (J.W.); haoran_jia@163.com (H.J.); wjf266@163.com (J.W.); 2 Hebei Key Laboratory of Electric Machinery Health Maintenance & Failure Prevention, North China Electric Power University, Baoding 071003, China; 3 School of Nuclear Science and Engineering, North China Electric Power University, Beijing 102206, China

**Keywords:** diamond, graphite, Al matrix composites, reactive wetting

## Abstract

In this work, graphite and diamond are successfully introduced into Al melts and TiC is in situ-synthesized based on reactive wetting. It is found that the microstructures of the prepared TiC-reinforced Al composites are varied with the change in carbon sources and their sizes. TiC particles tend to form agglomerations in the composites prepared by both graphite and diamond, but the size of the TiC particles as well as their agglomerations will decrease with the decrease in the carbon source size. In addition, the Ti-C reaction is also difficult to fully carry out due to the influence of the Al-C reaction. As a result, in addition to TiC particles, Al_4_C_3_ will also be present in the composites prepared by graphite, especially when the size of graphite is large. As for the composites prepared by diamond, diamond@Al_4_C_3_@TiC core–shell particles will form when the size of the diamond is large, such as 10 μm in this work, and these particles will transform into Al_4_C_3_@TiC core–shell particles when the size of the diamond is decreased.

## 1. Introduction

Metal matrix composites (MMCs) are types of materials that are based on metal and its alloy, and they are artificially combined with one or more metal or nonmetal-reinforced phases. Among them, particle-reinforced Al matrix composites have been widely studied and used in aerospace structural parts, automotive and military equipment, the electronic industry, and other fields due to their light weight, high specific elastic modulus, high specific strength, wear resistance, low coefficient of thermal expansion, and excellent dimensional stability [1,2,3,4,5,6]. TiC has excellent properties such as high melting point, high modulus and hardness, good thermal conductivity and electrical conductivity, and low thermal expansion coefficient. It has been widely used in many fields, such as machinery, chemistry, and microelectronics. Therefore, it is usually used as the reinforcement particles for MMCs [7,8,9,10,11].

Because of the poor wettability between TiC and most metals, how to realize the composite of TiC and the metal, and form a good interface is the key in the preparation of TiC-reinforced metal matrix composites. For the time being, a variety of MMC preparation methods have been developed, such as spark plasma sintering (SPS), melt infiltration (MI), and the contact reaction method (CRM) [12,13,14,15]. In the contact reaction method, the reaction element powder is made into a preformed block, the reinforcement phase is in situ-synthesized in the high-temperature metal melt, and the composites are finally obtained by casting. This method has the advantages of low cost, simple processes, and good bonding between the reinforcement and matrix, and the composites with the various shapes and sizes can especially be obtained by casting [16,17,18,19].

The contact reaction method has also been used to prepare TiC-reinforced Al matrix composites, in which Ti and C sources are separately or together added into Al melts to in situ-synthesize TiC. However, due to the interface contact angle between most carbon sources, such as graphite, and Al melts being much more than 90°, which means poor wettability, the carbon sources are also difficult to introduce into Al melts [20,21,22,23,24,25]. In addition, it is found that the microstructure uniformity of TiC synthesized by CRM, including the uniformity of its particle size and distribution, is usually poor [23,24,25]. The above factors limit the preparation of TiC-reinforced Al matrix composites by the CRM.

In our recent studies, different carbon sources have been successfully introduced into Al and Cu melts based on the Ti-C reaction to in situ-synthesized TiC [26,27,28,29], and it has been found that the types of carbon sources, as well as their size, will result in a great difference in TiC microstructures [29,30]. Thus, understanding the influence of carbon sources on the microstructures of TiC and its mechanism is conducive to better controlling the microstructures of the TiC-reinforced Al matrix composites prepared by CRM.

Therefore, in this work, graphite and diamond with different sizes have been used to in situ-synthesize TiC by the Ti-C reaction in Al melts. The microstructures of TiC and its formation processes have been investigated.

## 2. Experimental

**Figure 1:**
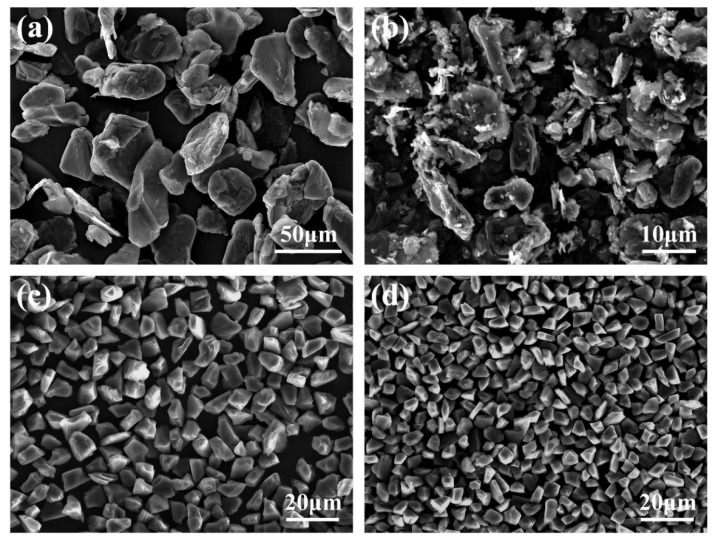
Microstructures of the used carbon sources: (**a**) 30 μm graphite, (**b**) 4 μm graphite, (**c**) 10 μm diamond, and (**d**) 6 μm diamond.

Sizes of 30 μm and 4 μm graphite, 10 μm and 6 μm diamond powder, 30 μm Ti powder, and industrial pure Al were used as raw materials to in situ-synthesize TiC in Al melts in this work. The microstructures of the used carbon sources are shown in Figure 1.

**Figure 2:**
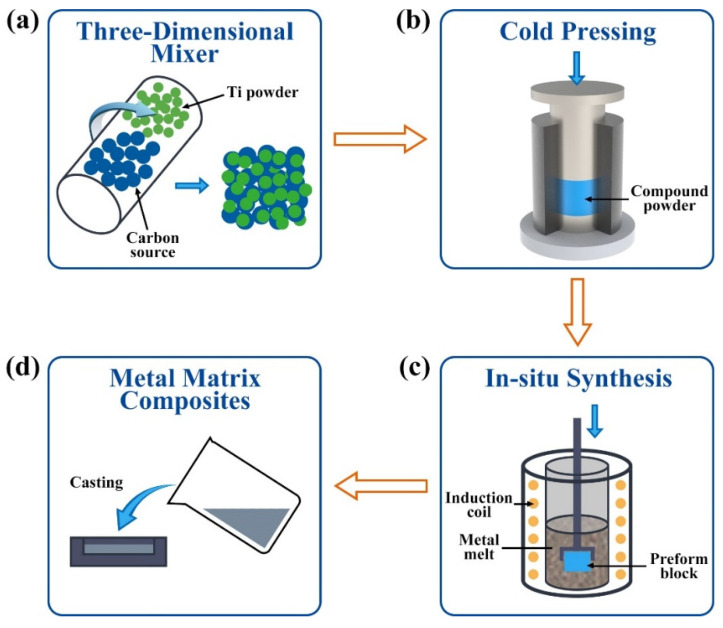
The preparation processes of Al-Ti-C composites. (**a**) mixing of the powders. (**b**) compaction o©he powders. (**c**) in-situ synthesis of TiC in Cu melting. (**d**) casting.

The TiC-reinforced Al matrix composites with the nominal composition of Al-4 wt.%Ti-1 wt.%C (it is herein designated as Al-4Ti-1C and the C in Al-4Ti-1C is a general term for graphite and diamond in this paper; when we need to specify that C is graphite or diamond, we write Al-4Ti-1C as Al-4Ti-1G or Al-4Ti-1D) are prepared. The composition of the composite was determined according to the stoichiometric ratio of TiC. In theory, if the carbon source completely reacts with Ti to synthesize TiC, 4% carbon and 1% Ti are needed. The preparation processes are shown in Figure 2. At first, the Ti powder and different carbon sources were weighed according to 4% and 1% of the total weight, respectively, and then the used powders were mixed in a three-dimensional mixer for 4 h. In order to better study the influence of carbon source on the microstructures of TiC, no other treatments were carried out on the Ti and carbon source mixed powder. Next, under the condition of temperature 20 °C and pressure 20 MPa, the mixed powder was pressed into a cylindrical preform block with a diameter of 25 mm, as shown in Figure 2b. Then, the preform was added into the Al melt at a temperature of about 1150 °C, as shown in Figure 2c. Finally, after holding for about 5 min, the melt was poured into the graphite mold to obtain the composites.

The prepared as-cast samples were then cut and polished by standard procedures and analyzed by scanning electron microscopy (SEM) equipped with energy-dispersive X-ray spectroscopy (EDS) and X-ray diffraction (XRD). The standard shape and size of the SEM analysis sample was a 10 mm high, 12 mm × 12 mm square column. In addition, in order to better analyze the microstructures of the TiC, the TiC powder that was extracted from the as-cast samples by absolute etching of the Al matrix in an etching solution was also analyzed by SEM.

## 3. Results and Discussion

**Figure 3:**
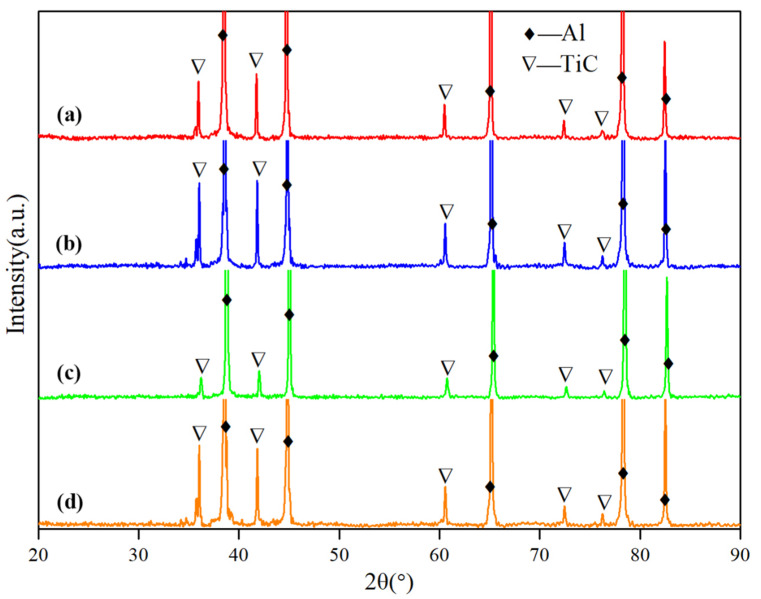
XRD results of as-cast Al-4Ti-1C prepared by different carbon sources: (**a**) 30 μm graphite, (**b**) 4 μm graphite, (**c**) 10 μm diamond, and (**d**) 6 μm diamond.

During the preparation processes, it was found that all the preforms with the different carbon sources react quickly after adding into the Al melt, and the reaction products can be dispersed in the Al melt. Figure 3 shows the XRD results of the obtained as-cast composites, and Figure 3a–d correspond to the results of XRD phase analysis of Al-4Ti-1C composites prepared by 30 μm graphite, 4 μm graphite, 10 μm diamond, and 6 μm diamond, respectively. It can be seen that TiC has been synthesized in all samples, which indicates that both graphite and diamond can be introduced into the Al melt by Ti-C reaction. Comparing the XRD patterns of the same carbon source under different sizes, we can see that the amount of TiC produced by the reaction of 4 μm graphite and 6 μm diamond with Ti in the aluminum melt is more than that produced by the reaction of 30 μm graphite and 10 μm diamond, indicating that the reduction in carbon source size is conducive to the full reaction of Ti and C. The amount of TiC produced by 10 μm diamond as a carbon source is much less than that of 4 μm diamond as a carbon source, indicating that the particle size has a greater influence on the reaction between diamond and Ti in the aluminum melt than that of graphite.

### 3.1. Microstructure and Distribution of TiC in Al Melt Synthesized by Graphite

**Figure 4:**
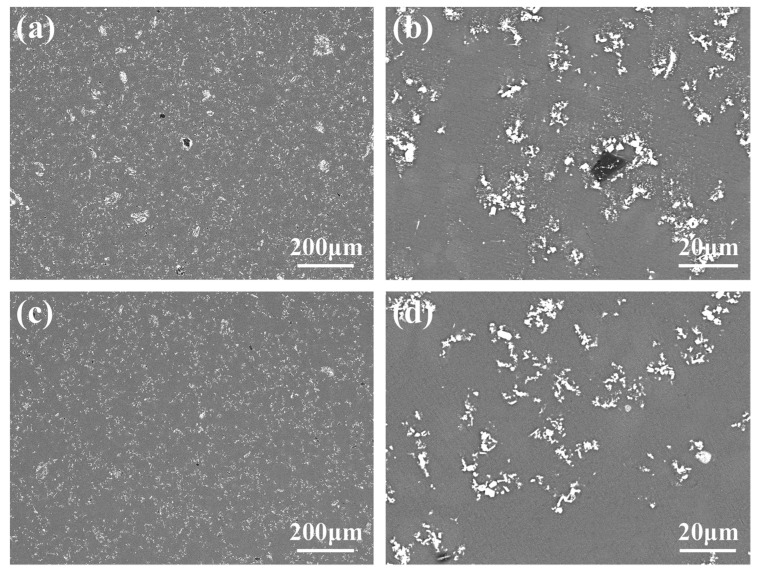
Microstructures of Al-4Ti-1C composites prepared by graphite with different sizes: (**a**,**b**) 30 μm graphite and (**c**,**d**) 4 μm graphite.

The microstructures of the Al-4Ti-1C composites prepared by 30 μm graphite (it is herein designated as Al-4Ti-1G (30 μm)) are shown in Figure 4a,b. It can be seen from Figure 4a that there are two different phases in the Al matrix. The main phase is white and particle-like. Most of them are dispersed on the Al matrix, while some of them have been gathered into agglomerations with sizes between 10 μm and 30 μm. Actually, it is found from the magnified microstructures shown in Figure 4b that the seemingly dispersed particles shown in Figure 4a are also agglomerations composed of several particles. The second phase is gray and block-like, which is usually surrounded by the white particles, as shown in Figure 4b.

**Figure 5:**
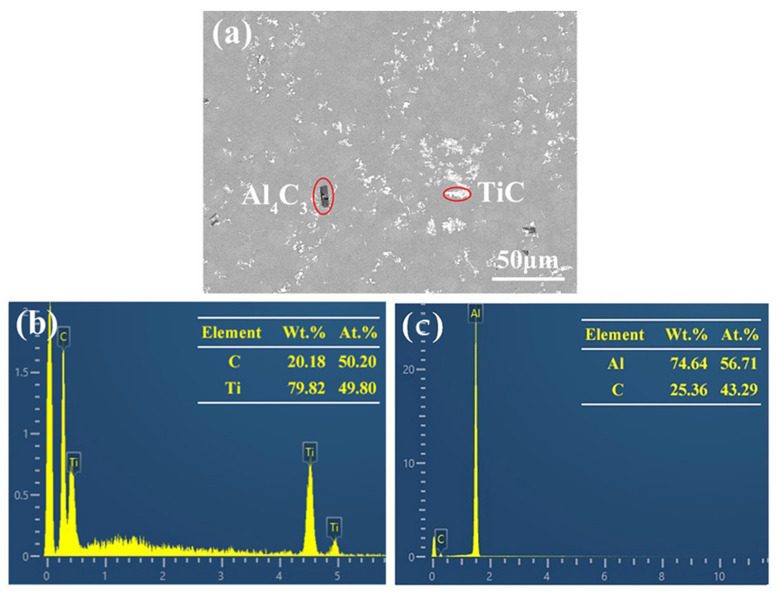
EDS point analysis of Al-4Ti-1C composites prepared by 30 μm graphite: (**a**) microstructure and (**b**,**c**) corresponding EDS point analysis results.

In order to clarify the composition of the above phases, EDS point analysis was carried out, and the results are shown in Figure 5. Figure 5b shows that the contents of the white particles are mainly Ti and C, in which the ratio of Ti/C is close to 1:1. Combined with the XRD analysis of Figure 3a, it is confirmed that the white particles are in situ-synthesized TiC. Figure 5c shows that the gray block phase is mainly composed of Al and C, and the ratio of Al/C is about 4:3, indicating that it is an Al_4_C_3_ compound. It is known that Al is easy to react with C at the high temperature, so the Al_4_C_3_ compound is proven.

**Figure 6:**
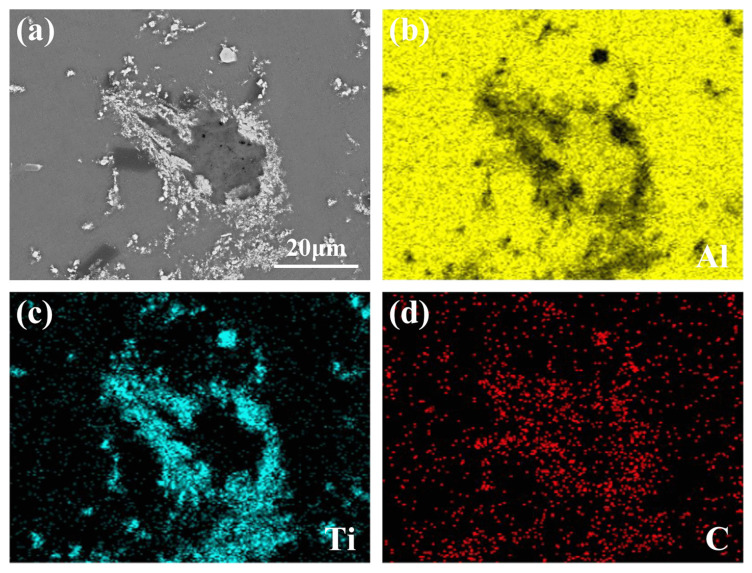
EDS mapping analysis of the core–shell phase of Al-4Ti-1C prepared by 30 μm graphite: (**a**) microstructure and (**b**–**d**) mapping micrographs for Al, Ti, and C elements.

Figure 6 shows the mapping analysis results of the typical core–shell phase. As can be seen from Figure 6b,d, the core of the phase is mainly composed of Al and C elements, further indicating it is Al_4_C_3_. Figure 6c,d show that the shell is mainly composed of Ti and C elements, which is determined to be the TiC shell. This kind of core–shell structure is proven to be Al_4_C_3_@TiC. The formation of Al_4_C_3_@TiC demonstrates that some graphite is not directly reacted with Ti to form TiC but first reacts with Al to form Al_4_C_3_, and then further reacts with Ti.

**Figure 7:**
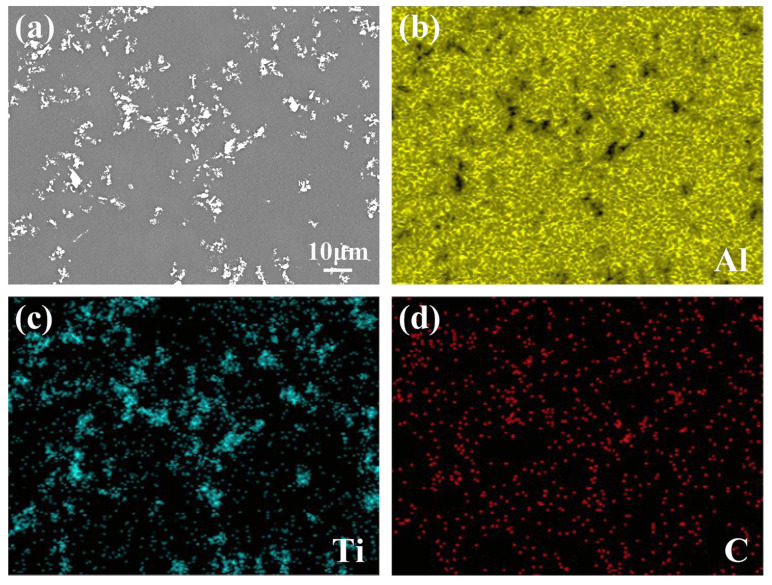
EDS mapping analysis of Al-4Ti-1C composites prepared by 4 μm graphite: (**a**) microstructure and (**b**–**d**) mapping micrographs for Al, Ti, and C elements.

Figure 4c,d show the microstructures of Al-4Ti-1C composites prepared by 4 μm graphite (it is herein designated as Al-4Ti-1G (4 μm)). Combined with the EDS mapping analysis results shown in Figure 7, it can be found that although the synthesized TiC particles still tend to form agglomerations, the size of the agglomeration is obviously reducible, and the distribution is more uniform. At the same time, it is found that the size range of the TiC is also effectively decreased.

**Figure 8:**
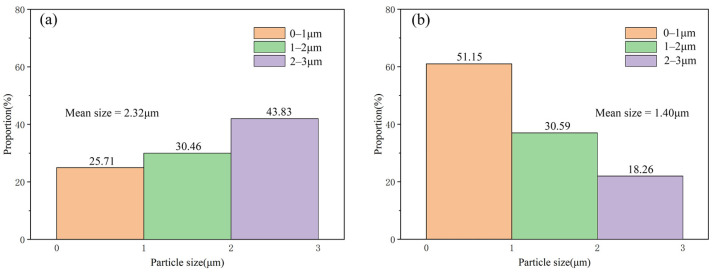
The size and its distribution of TiC in the composites prepared by different graphite sizes: (**a**) 30 μm graphite and (**b**) 4 μm graphite.

It can be seen from Figure 8 that the mean size of TiC particles in Al-4Ti-1G (30 μm) is about 2.32 μm, while that in Al-4Ti-1G (4 μm) is decreased to about 1.40 μm. In addition, it is found that there is almost no Al_4_C_3_ in the Al matrix, demonstrating that the reaction between Ti and 4 μm graphite is more sufficient than that between Ti and 30 μm graphite.

**Figure 9:**
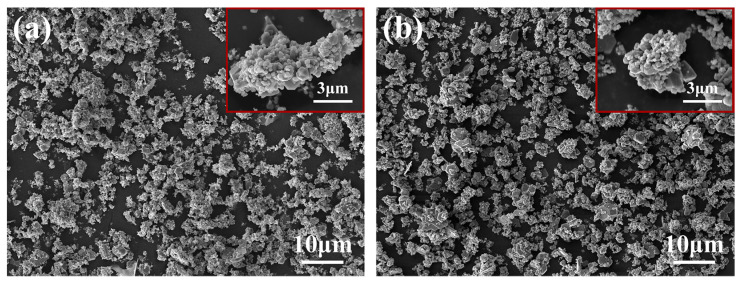
Microstructures of the extracted TiC from the as-cast samples: (**a**) Al-4Ti-1G (30 μm) and (**b**) Al-4Ti-1G (4 μm).

In order to further observe the morphology of the synthesized TiC, the TiC was extracted from the as-cast sample and the microstructures are shown in Figure 9. The results further demonstrate that the TiC synthesized in Al-4Ti-1G (30 μm) and Al-4Ti-1G (4 μm) composites are mainly distributed in the form of agglomerations, rather than dispersed particles. From Figure 9a,b, it is found that the size of the agglomerations is close to the size of the original graphite. As a result, the size of the synthesized TiC agglomerations decreases with the decrease in the graphite particle size. From the enlarged image inset in Figure 9a,b, it is further found that the size of the TiC particles that make up the agglomerations varies greatly, from submicron to micron, which should correspond to the distribution of very small TiC particles in the TiC shell of Figure 6a.

According to the above results, it can be concluded that graphite can be effectively introduced into Al melts by Ti-C reaction to in situ-synthesize TiC. Reducing the particle size of graphite is beneficial to promote the full reaction of Ti-C and improve the distribution of TiC.

### 3.2. Microstructure and Distribution of TiC in Al Melt Prepared by Diamond

**Figure 10:**
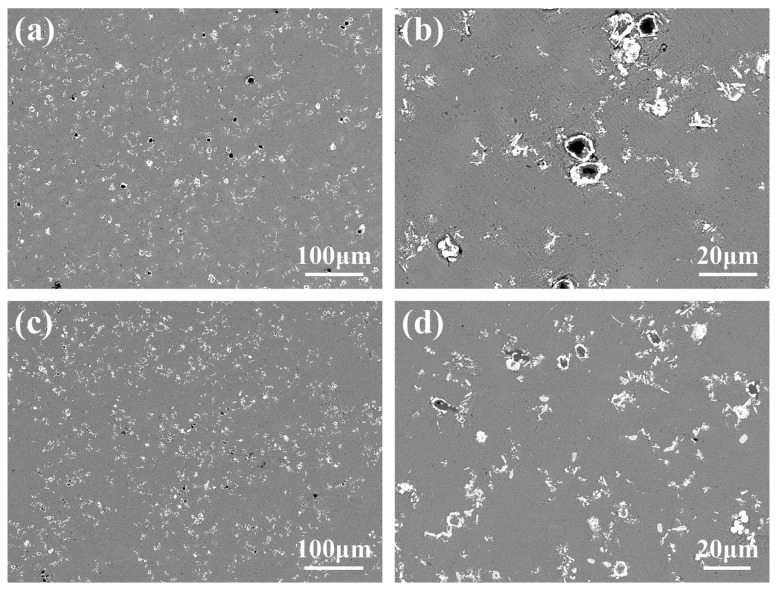
Microstructures of Al-4Ti-1C composites prepared by diamond with different sizes: (**a**,**b**) 10 μm diamond and (**c**,**d**) 6 μm diamond.

In this section, diamond is used as a carbon source to react to synthesize TiC in the Al melt. Figure 10a,b show the microstructures of as-cast samples of Al-4Ti-1C composites prepared by 10 μm diamond (it is herein designated as Al-4Ti-1D (10 μm)), while Figure 10c,d show the microstructures of the as-cast samples of Al-4Ti-1C composites prepared by 6 μm diamond (it is herein designated as Al-4Ti-1D (6 μm)). It can be seen from Figure 10 that the microstructure of the composites is different from the Al-4Ti-1C prepared by the graphite shown in Figure 4. Both white particles, which are the synthesized TiC, and many core–shell particles whose size is almost the same as that of the used diamond were synthesized in the Al matrix.

**Figure 11:**
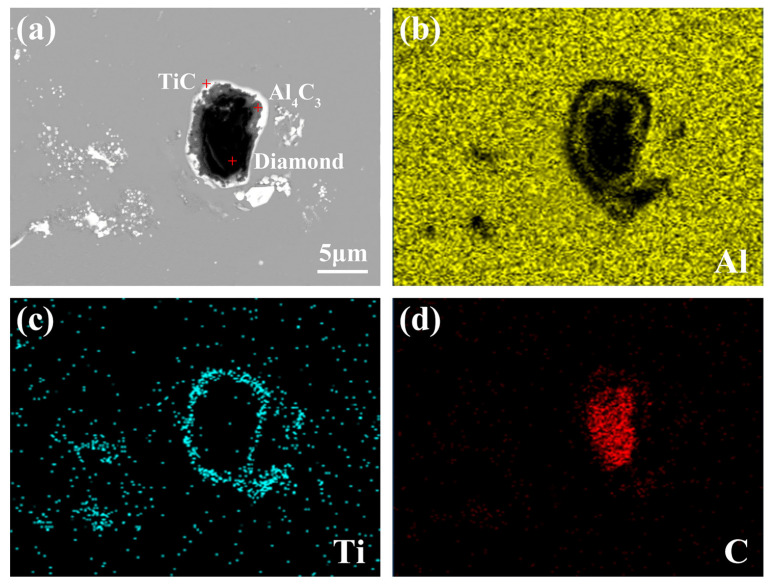
EDS mapping analysis of core–shell particles in Al-4Ti-1D (10 μm): (**a**) microstructure and (**b**–**d**) mapping micrographs for Al, Ti, and C elements.

Figure 11a shows that the core–shell particles in Al-4Ti-1D (10 μm) have a three-layer structure, where the outermost layer is a dense white phase, the core is a black phase, and there is a layer of gray phase in the middle. It is found from Figure 11b,d that the outermost white phase mainly contains Ti and C elements, indicating that it should be a TiC shell. The middle layer mainly contains Al and C elements, which correspond to Al_4_C_3_. There is only the C element in the core phase, which is an unreacted diamond. Thus, this core–shell structure should be diamond@Al_4_C_3_@TiC core–shell particles. The results demonstrate that 10 μm diamond can also be introduced into the Al melt to synthesize TiC particles. However, due to the formation of the TiC shell on the diamond surface in some cases, the reaction between Ti and diamond is difficult to fully carry out, which results in the formation of diamond@Al_4_C_3_@TiC core–shell particles. This finding is in agreement with the results of our previous work [27,28].

**Figure 12:**
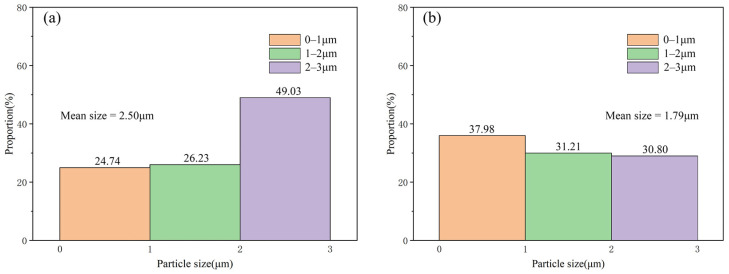
The size and its distribution of TiC in the composites prepared by different diamond sizes: (**a**) 10 μm diamond and (**b**) 6 μm diamond.

It can be seen from Figure 10c that, compared with Al-4Ti-1D (10 μm), the number of core–shell particles in Al-4Ti-1D (6 μm) is decreased, while the TiC particles are more dispersed. In addition, the mean size of the TiC also decreases from about 2.5 μm to 1.79 μm with the decrease in the diamond size, as shown in Figure 12.

**Figure 13:**
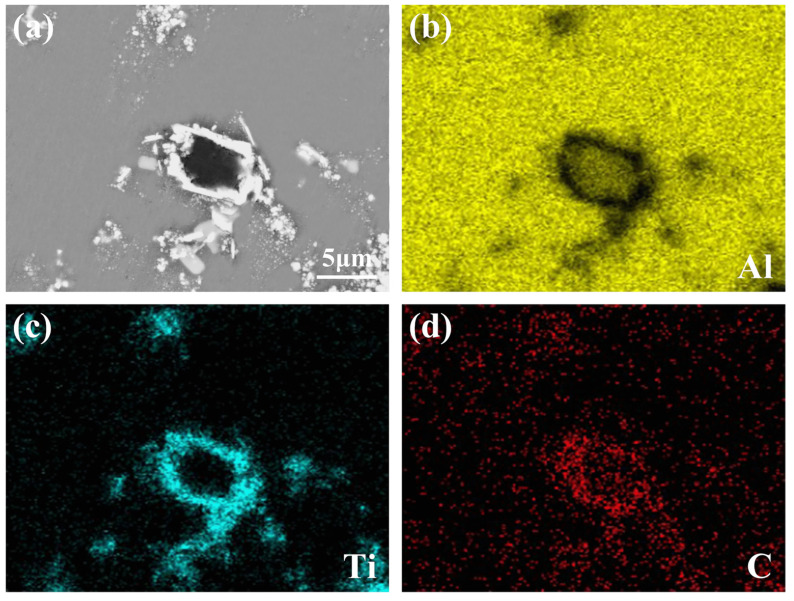
EDS mapping analysis of core–shell particles in Al-4Ti-1D (6 μm): (**a**) microstructure and (**b**–**d**) mapping micrographs for Al, Ti, and C elements.

Figure 10d shows that although a few of the core–shell particles in the Al-4Ti-1D (6 μm) have a three-layer structure, most of them have a two-layer structure, which has a white shell and gray core. In order to determine the composition of the two-layer core–shell particles, the EDS mapping analysis was carried out and the results are shown in Figure 13. It can be seen that the core phase mainly contains Al and C, indicating that it is Al_4_C_3_. Figure 13c,d show that the shell mainly contains Ti and C. Thus, the two-layer core–shell particle should be Al_4_C_3_@TiC.

**Figure 14:**
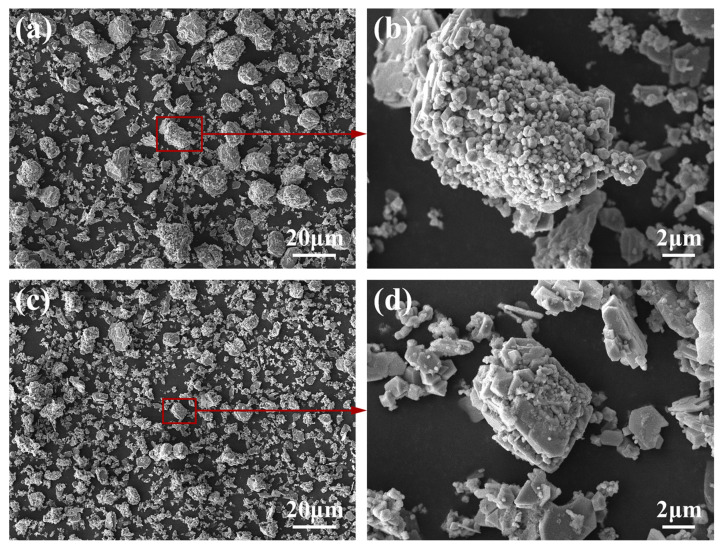
Microstructure morphology of extracted core–shell particles: (**a**,**b**) Al-4Ti-1D (10 μm) and (**c**,**d**) Al-4Ti-1D (6 μm).

The microstructures of the powder extracted from the as-cast samples are shown in Figure 14. The core–shell particles in Figure 14a,c correspond to the core–shell particles in Figure 11a and Figure 13a, respectively. From Figure 14b,d, the core–shell particle shell of Al-4Ti-1D (10 μm) is composed of small dense TiC particles, while the shell of the core–shell particle in Al-4Ti-1D (6 μm) is composed of larger TiC particles, indicating that more TiC particles can nucleate and grow on the surface of diamond with the increase in its size and tend to form a denser TiC shell, which will prevent the further Ti-C reaction. This may be the reason that Al-4Ti-1D (10 μm) has more core–shell diamond@Al_4_C_3_@TiC particles.

The above results show that the size distribution of synthesized TiC particles by diamond is like that of graphite. With the decrease in diamond size, the reaction between diamond and Ti in the Al melt is more sufficient, and the microstructure is more uniform. However, the reaction products are very different from the composites prepared by the graphite. Not only are TiC particles formed, but TiC shells will also more easily form on the surface of the diamond, which will prevent the full reaction of Ti-C and then form diamond@Al_4_C_3_@TiC or Al_4_C_3_@TiC core–shell particles.

### 3.3. The Synthesis Processes of TiC in Al Melts Used Different Carbon Sources

According to the current research results, when the Ti-C preform block is added to the aluminum melt, the possible reactions are as follows [27]:

(1) Ti+C → TiC

(2) $$\mathrm{Al}+C\;\to {\;\mathrm{Al}}_{4}C_{3}$$

(3) $${\mathrm{Al}}_{4}C_{3}+\mathrm{Ti}\;\to \;\mathrm{TiC}+\mathrm{Al}$$

In the previous study, we also tried to add graphite or diamond directly into the Al melt or Al-Ti melt by stirring. However, due to the poor wettability of the carbon source with the Al and Al-Ti melt, it was found that most of the graphite and diamond floated and burned out after adding to the melt, so it was difficult to introduce into the Al melt. The comparative analysis shows that the introduction of graphite/diamond in this study is mainly attributed to the rapid reaction (1) of the Ti-C mixed preform block, thus realizing the reaction wetting between graphite/diamond and Al. After reducing the particle size of the same carbon source, the microstructure uniformity of the composite is improved, indicating that reducing the particle size of the carbon source is helpful to promote the reaction between Ti-C and the uniform distribution of the synthesized products. The change in carbon source leads to the change in the type of reaction products in the melt. Compared with the composites prepared by graphite, not only particles and clusters but also core–shell particles are formed in the composites prepared by diamond.

As shown in Figure 1, it is found that the graphite is mainly flaky particles, while the diamond is polyhedral particles. When the preform is added into the Al melt, the flaky graphite particles are more likely to react fully, while the full reaction of polyhedral diamond takes longer. This will lead to the formation of a dense TiC shell on the diamond surface, thus preventing the Ti-C reaction. This is the reason why core–shell particles are easy to form when diamond is used to prepare composite materials. The shells of the core–shell particles synthesized in the composite materials are all TiC, and there are two reasons for the formation of a TiC shell: (i) It is reported that the atomic diffusion rate is related to the radius, and the diffusion rate of Al is faster than that of Ti. When the carbon source reacts with the mixed melt of Al-Ti, the Al-C reaction tends to be inside the carbon source, while the Ti-C reaction occurs on the surface of the carbon source [31]. (ii) Because TiC is more thermodynamically stable, Al_4_C_3_ will further react with Ti by reaction (3) to form TiC. The TiC formed at the same time is easier to nucleate and grow on the surface of Al_4_C_3_, thus forming a TiC shell.

**Figure 15:**
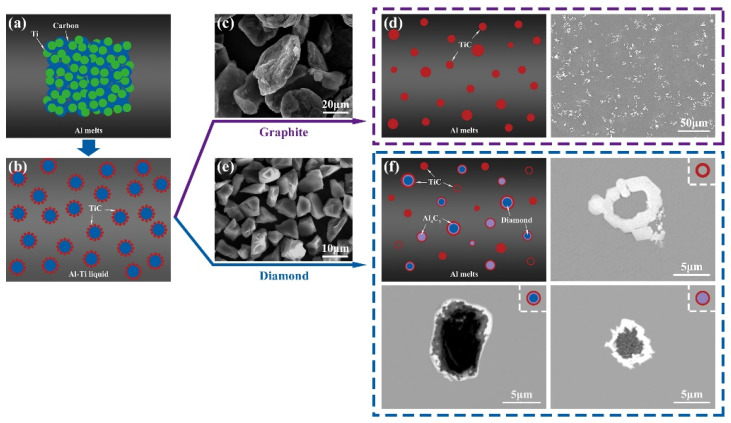
Synthesis processes of TiC after adding the Ti-C mixture into Al melts: (**a**) adding Ti-C mixture into Al melt, (**b**) TiC synthesized on the surface of carbon sources, (**c**) the used graphite, (**d**) the formed TiC clusters, (**e**) the used diamond, and (**f**) the formed TiC and core–shell particles.

Furthermore, compared with the core–shell particles prepared by different sizes of diamond, it is found from Figure 14b,d that, in addition to the different sizes, the morphology of the TiC in the shell of Al-4Ti-1D (10 μm) and Al-4Ti-1D (6 μm) is also very different. As shown in Figure 14b, all the TiC in the shell of Al-4Ti-1D (10 μm) is particle-like with almost the same size, while some plate-like TiC is formed in the shell of Al-4Ti-1D (6 μm), and the size of particle-like TiC also has a large difference, as Figure 14d shows. It has been reported that TiC formed by Al_4_C_3_ and Ti reactions is easy to become plate-like [32,33]. Therefore, it is considered that the core–shell particles prepared from 6 μm diamond have simultaneous Al-C and Ti-C reactions on the diamond surface. Therefore, Al_4_C_3_ and TiC will be formed on the diamond surface. The formed Al_4_C_3_ will prevent the TiC particles from forming a dense shell structure, so the reaction of 6 μm diamond is more sufficient. Finally, the Al_4_C_3_ on the surface will further react with Ti to form flaky TiC and the inner diamond will become Al_4_C_3_, which will result in the formation of Al_4_C_3_@TiC core–shell particles. However, when the preform block prepared by 10 μm diamond is added to the Al melt, the reactivity of Al-C and Ti-C decreases due to the increase in particle size. As mentioned above, the reason why diamond can be introduced into the melt is due to the reactive wetting of Ti-C. Therefore, the 10 μm diamond in the melt tends to be a Ti-C reaction, and more TiC particles are formed on the diamond surface to form a dense shell. Therefore, more diamond@Al_4_C_3_@TiC particles were formed in the composites prepared in Al-4Ti-1D (10 μm) than prepared in Al-4Ti-1D (6 μm). The whole process of synthesizing and growing TiC with different morphologies by adding preform blocks into Al melts is illustrated in Figure 15.

## 4. Conclusions

To sum up, based on the Ti-C reaction, the graphite/diamond can be effectively introduced into the Al melt to prepare TiC-reinforced composites, and the microstructures of TiC generated by the reaction change with the change in carbon sources and their sizes, which lays a good foundation for the preparation of TiC-reinforced aluminum matrix composites. It is found that TiC particles tend to form agglomerations in the composites prepared by both graphite and diamond, but the size of the TiC particles as well as their agglomerations will decrease with the decrease in the carbon source size. In addition, the Ti-C reaction is also difficult to fully carry out due to the influence of the Al-C reaction. As a result, in addition to TiC particles, Al_4_C_3_ will also be present in the composites prepared by graphite, especially when the size of graphite is 10 μm. As for the composites prepared by diamond, diamond@Al_4_C_3_@TiC core–shell particles will be formed when the size of diamond is 10 μm, and these particles will be transformed into Al_4_C_3_@TiC core–shell particles when the size of the diamond is 6 μm. Al diffuses faster than Ti in diamond, so Al diffuses into diamond faster and reacts with diamond to form Al_4_C_3_. On the surface of diamond, because TiC is more thermodynamically stable, Al_4_C_3_ will further react via Ti reaction to form TiC. When TiC grows to form a dense shell, it prevents the further diffusion of Ti and Al in diamond, thus forming a core–shell structure.

## Data Availability

The data support the finding of this study are available from the corresponding author, Fang Liu, upon reasonable request.
